# Retraction: Functional disruption of staphylococcal accessory regulator A from *Staphylococcus aureus* by silver ions

**DOI:** 10.1039/d1ra90084f

**Published:** 2021-02-25

**Authors:** Xiangwen Liao, Guijuan Jiang, Jing Wang, Jintao Wang

**Affiliations:** School of Pharmacy, Jiangxi Science & Technology Normal University Nanchang 330013 China liao492008522@163.com +86 791 8380 2393 +86 791 8380 2393

## Abstract

Retraction of ‘Functional disruption of staphylococcal accessory regulator A from *Staphylococcus aureus* by silver ions’ by Xiangwen Liao *et al.*, *RSC Adv.*, 2020, **10**, 33221–33226. DOI: 10.1039/D0RA06357F.

We, the named authors, hereby wholly retract this *RSC Advances* article as there is an error in the data and the conclusions of the paper are not supported.

Fig. 3B does not show the titration data of SarA, it is the titration data of a different protein, and therefore we cannot unequivocally conclude that SarA binds Ag^+^*via* its conserved cysteine residues.

The corresponding authors regret this oversight and apologise for any inconvenience to readers.

Signed: Xiangwen Liao, Guijuan Jiang, Jing Wang and Jintao Wang

Date: 17^th^ February 2021

Retraction endorsed by Laura Fisher, Executive Editor, *RSC Advances*

## Supplementary Material

